# Supplementary figures and images for: Dual RNA-seq reveals a type 6 secretion system-dependent blockage of TNF-α signaling and BicA as a Burkholderia pseudomallei virulence factor important during gastrointestinal infection
Source: Gut Microbes. 2022 Aug 19;14(1):2111950. doi: 10.1080/19490976.2022.2111950 (PMC9397134; doi:10.1080/19490976.2022.2111950)

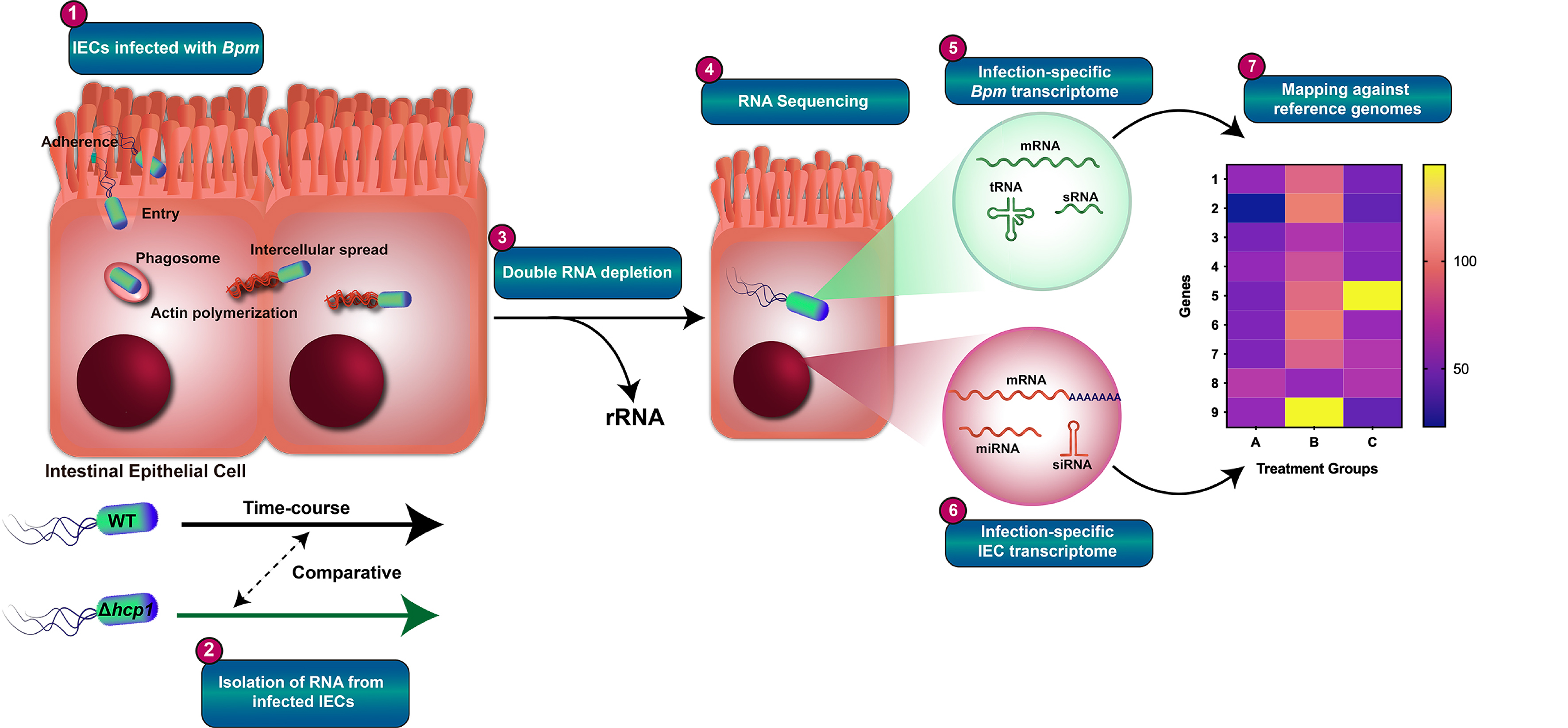

Supplement: Supplemental Material [file KGMI_A_2111950_SM1562.zip › Figure 1 modified.jpg]

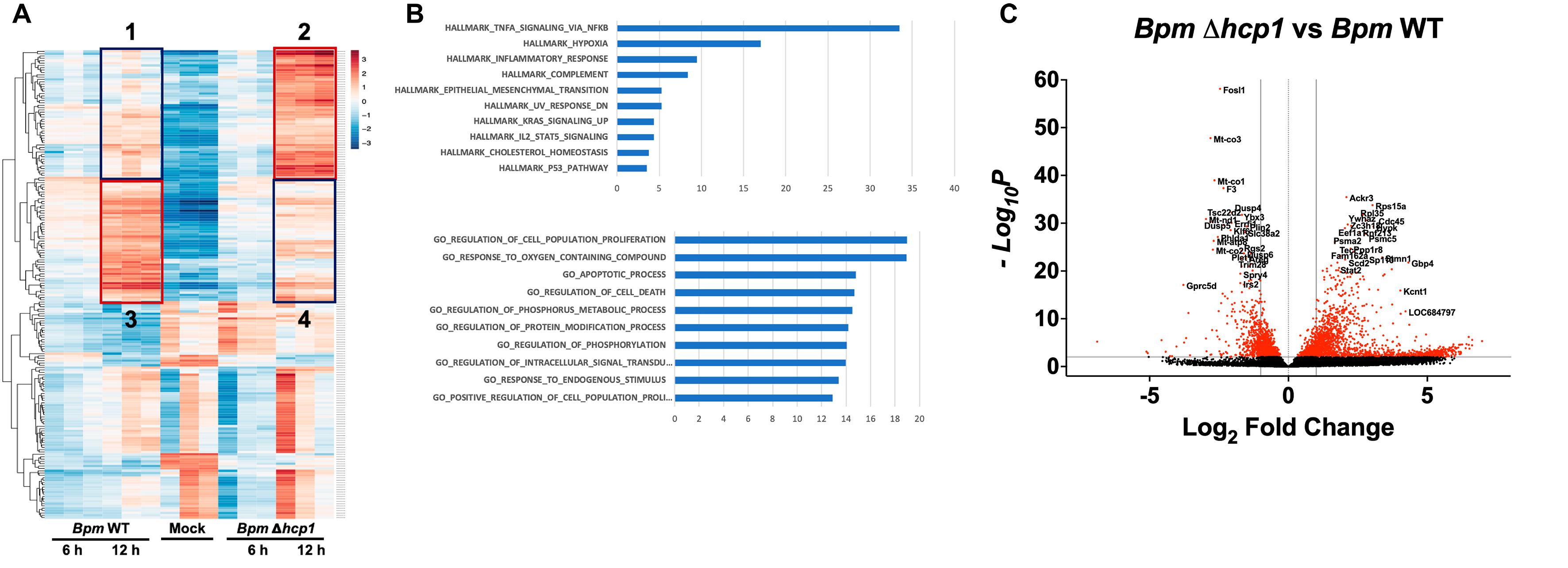

Supplement: Supplemental Material [file KGMI_A_2111950_SM1562.zip › Figure 2 modified.jpg]

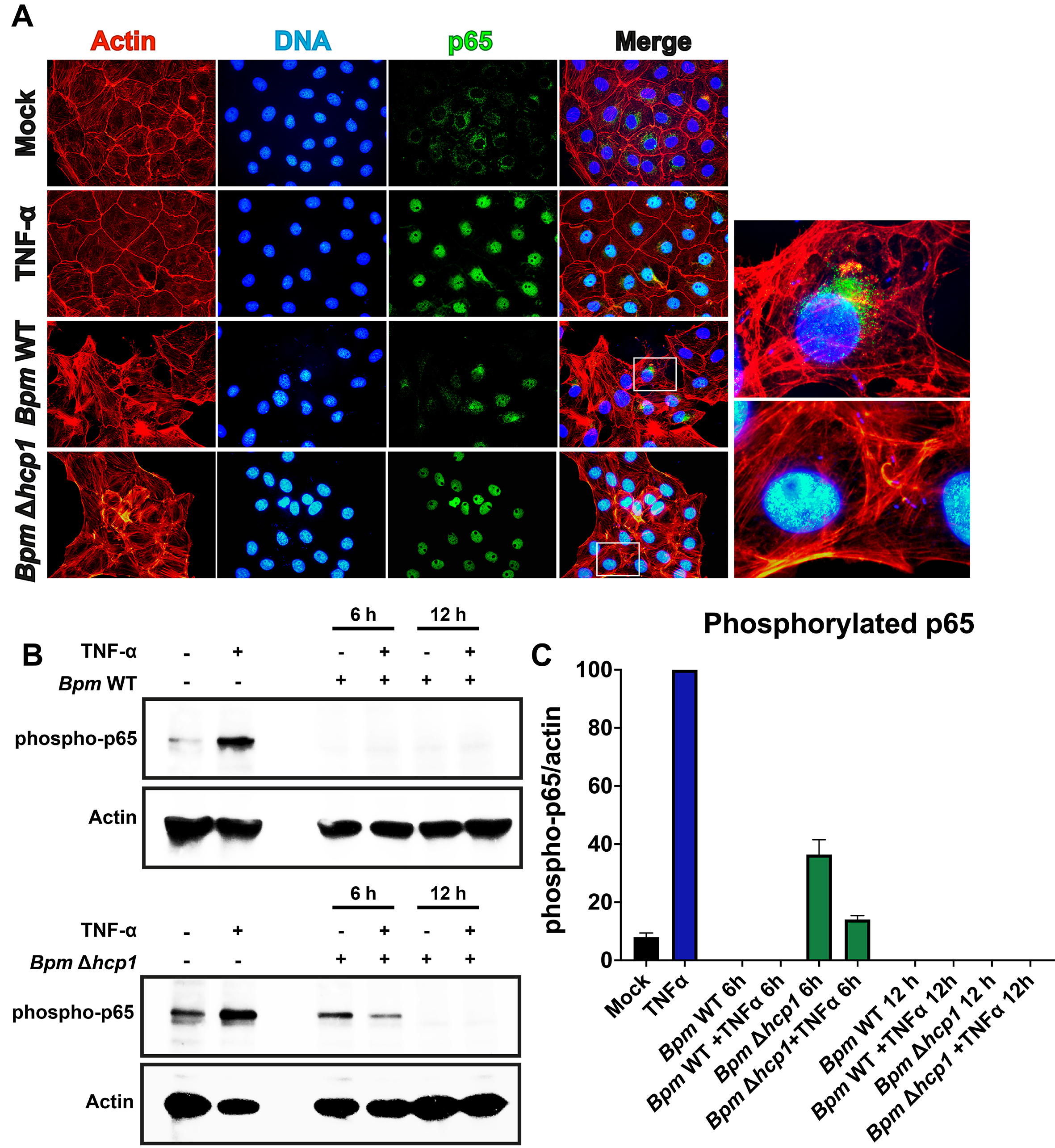

Supplement: Supplemental Material [file KGMI_A_2111950_SM1562.zip › Figure 3.jpg]

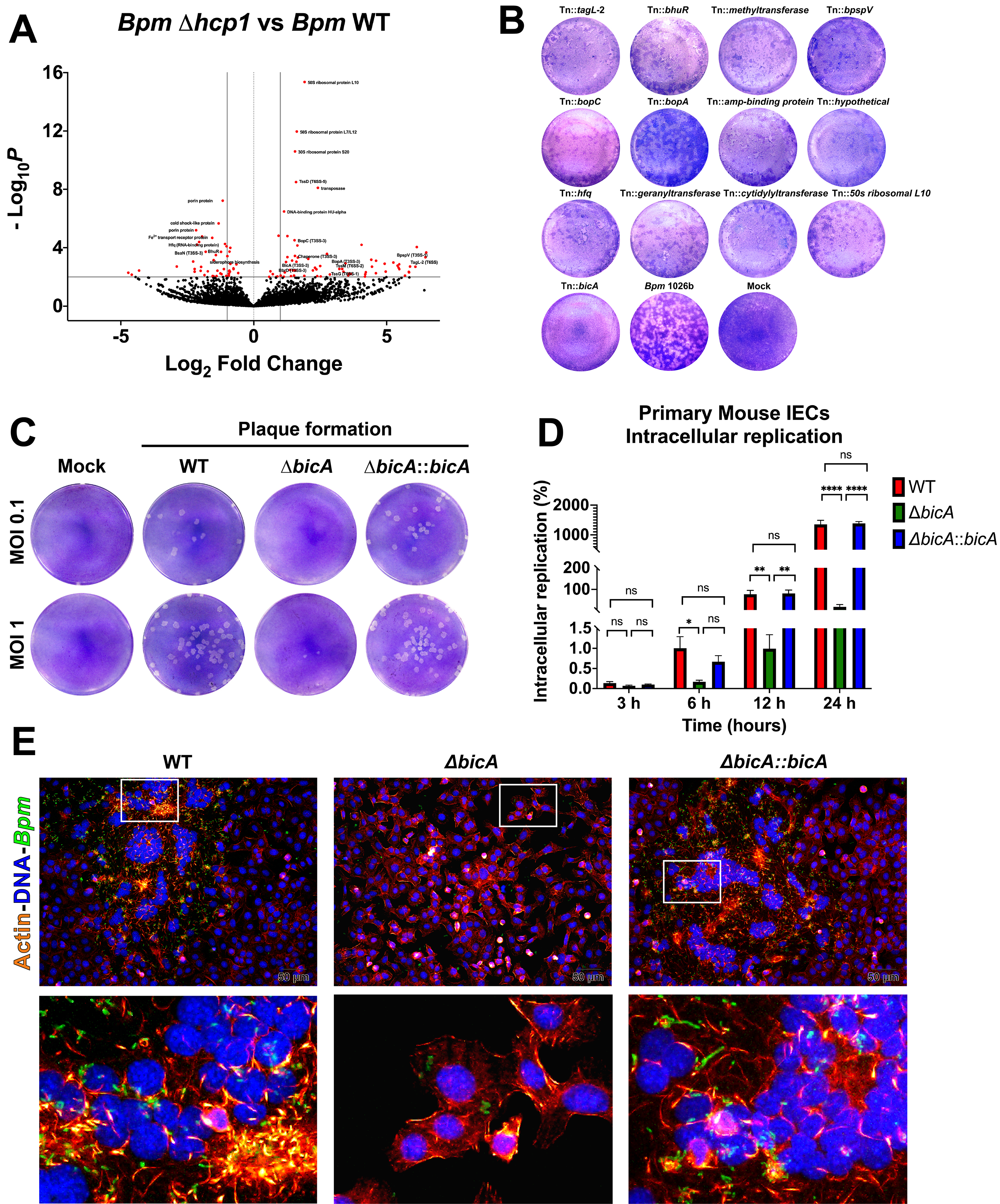

Supplement: Supplemental Material [file KGMI_A_2111950_SM1562.zip › Figure 4 modified.jpg]

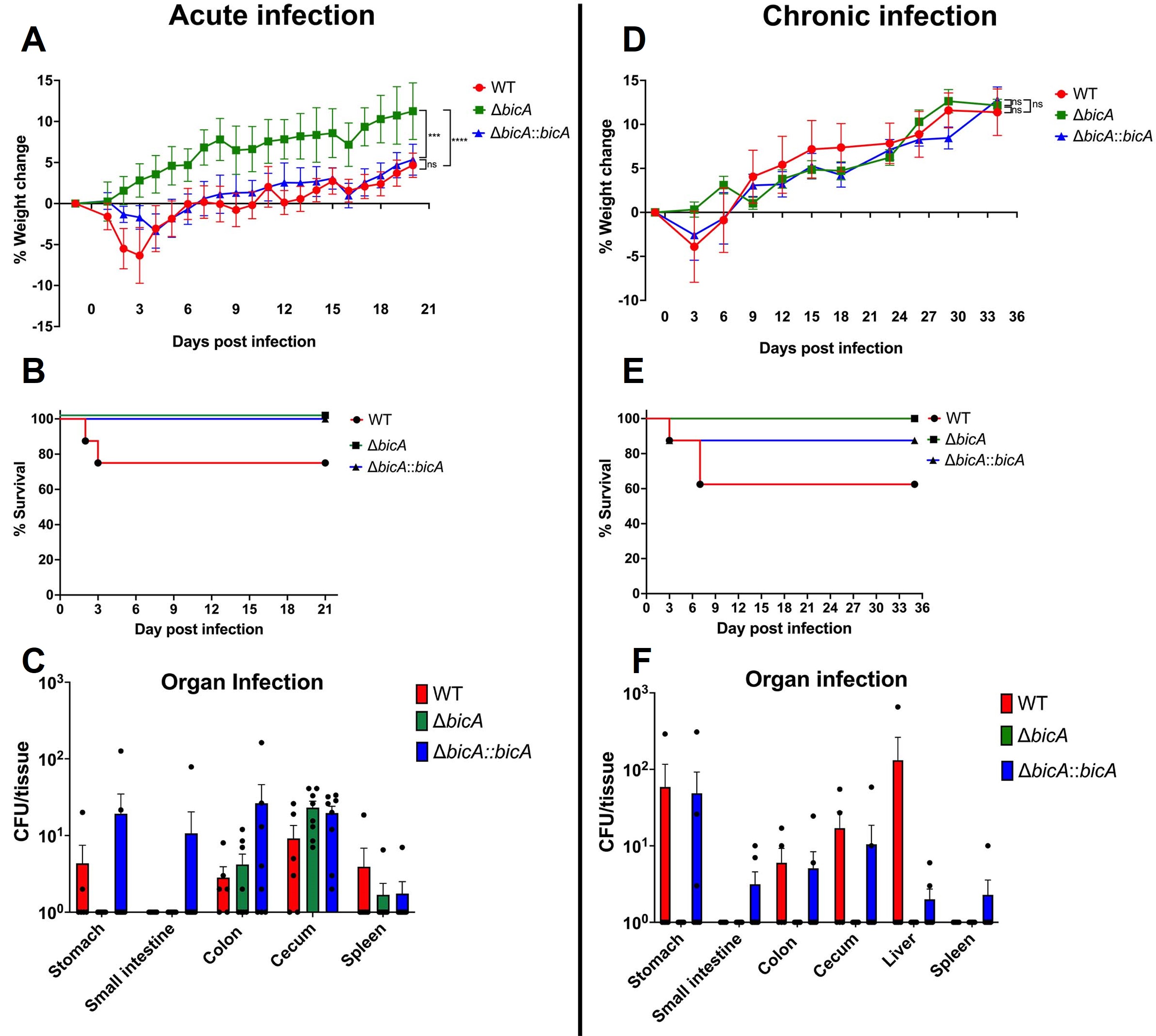

Supplement: Supplemental Material [file KGMI_A_2111950_SM1562.zip › Figure 5.jpg]
